# Clinical risk factors for new-onset atrial fibrillation in acute myocardial infarction: A systematic review and meta-analysis.

**DOI:** 10.1097/MD.0000000000015960

**Published:** 2019-06-28

**Authors:** Jing He, Yi Yang, Gui Zhang, Xiu-Hong Lu

**Affiliations:** a Guangdong Sanjiu Brain Hospital; b Guangdong Provincial Corps Hospital of Chinese People's Armed Police Forces, Guangzhou Medical University, Guangzhou, China.

**Keywords:** acute myocardial infarction, age, atrial fibrillation, heart rate

## Abstract

New-onset atrial fibrillation (NOAF) remains common arrhythmia in acute myocardial infarction (AMI), and is closely associated with increased subsequent cardiovascular mortality. Our meta-analysis aims to summarize more clinical risk factors for NOAF.

Comprehensive systematic search of MEDLINE, EMBASE, and the Cochrane Library were carried out to find relevant studies inception to December 2017. Pooled mean difference (MD) and 95% confidence interval (CI) were calculated to evaluate the value of clinical risk factors in the prediction of NOAF after AMI.

Eleven studies containing 9570 patients were included in the meta-analysis. Overall, older age and increased heart rate (HR) levels had a significant positive association with NOAF in patients with AMI. The MD in age between the patients with, and those without NOAF, was 8.22 units (95% confidence interval [CI]: 7.44–9.01), test for overall effect *z* score = 20.51 (*P* < .00001, *I*^2^ = 0%). Moreover, the MD in a subgroup analysis for HR levels between the patients with, and those without NOAF was 4.34 units (95% Cl: 2.56–6.11), test for overall effect *z* score = 4.78 (*P* < .00001, *I*^2^ = 31%).

In patient with AMI, our meta-analysis demonstrated that older age and increased HR levels on admission are related to greater risk of NOAF.

## Abstract 2

Supplemental Digital Content is available in the text

## 1 Introduction

Acute myocardial infarction (AMI) remains one of the leading causes of death globally. In spite of the widespread use of contemporary therapies, new-onset atrial fibrillation (NOAF) remains common arrhythmia in AMI, and is closely associated with considerable worse prognosis including prolonged hospitalization and all-cause mortality.^[1–5]^ Therefore, the identification of clinical risk factors related to NOAF in AMI is an important goal. Previous studies have demonstrated several risk factors related to NOAF, such as C-reactive protein, N-terminal pro-brain natriuretic peptide, CHADS2 score, high sensitivity troponin T, left ventricular ejection fraction, left atrium diameter, and obesity among others.^[6–11]^ However, the definite risk stratification of NOAF in AMI remains uncertain, and the aim of our systematic review and meta-analysis is to summarize more clinical risk factors for NOAF. To our knowledge, only a few studies directly evaluated the associations between age or heart rate (HR) and NOAF in patients with AMI. So we conducted this comprehensive meta-analysis to explore the impact of age or HR on NOAF following AMI by collecting data for previously published studies. Besides, the relationship of systolic blood pressure (SBP) or diastolic blood pressure (DBP) and NOAF was also assessed.

## 2 Methods

### 2.1 Identification of studies

A comprehensive systematic search of MEDLINE, EMBASE, and the Cochrane Library were carried out to find relevant studies inception to December 2017. The medical subject heading (MeSH) and text words for the term age or HR were combined with the MeSH term atrial fibrillation and AMI. Reference lists from the identified articles were manually examined for relevant new articles. Non-English language articles were not included.

### 2.2 Selection criteria

Abstracts and titles of related articles were initially scanned by a reviewer. Potentially relevant articles were then considered by at least 2 independent reviewers. Disagreements were resolved by discussion or upon consensus from a 3rd or 4th reviewer. Two reviewers agreed on the inclusionary or exclusionary status of 90% of the reviewed studies. Full texts of the selected articles were then screened by both authors for inclusion in the review. All disagreements were resolved by consensus. The included studies for analyses had to meet the following criteria: they were observational studies which include patients with AMI, which was defined as chest pain, elevated creatine kinase-MB or troponin level, and changed electrocardiogram according to guidelines; mean and standard deviation of age were reported; they used NOAF rates as an outcome; they were approved for the investigational review committee on human research. The exclusion criteria were: study included patients with a history of persistent or paroxysmal AF; studies were not published in English; abstracts without the full text.

### 2.3 Quality assessment and data extraction

Quality assessments were evaluated with the Newcastle-Ottawa Scale (NOS) list for nonrandomized studies. Each included study was in 3 aspects using this “star system”: the selection of the study groups; the comparability of the groups; and the ascertainment of the outcome of interest (Supplementary Material).

### 2.4 Statistical analysis

All analyses were conducted with the use of Review Manager, version 5.3 (Revman, The Cochrane Collaboration; Oxford, UK). The association strength between variable and NOAF was measured by mean difference (MD) and 95% confidence interval (CI). The significance of pooled MD was tested by *z* test (*P* < .05 was considered significant). Heterogeneity was evaluated with Cochran Q statistic and quality by *I*^2^ statistic. We premeditated that mild heterogeneity might be <30% percent of the variability in point estimates and the values of *I*^2^ exceeding 50% might be expressed as significant heterogeneity, so we considered to use the random-effects model for study, if not, use a fixed-effects model. Publication bias was also evaluated by inspecting funnel plots.

## 3 Results

### 3.1 Study Characteristics

**Figure 1:**
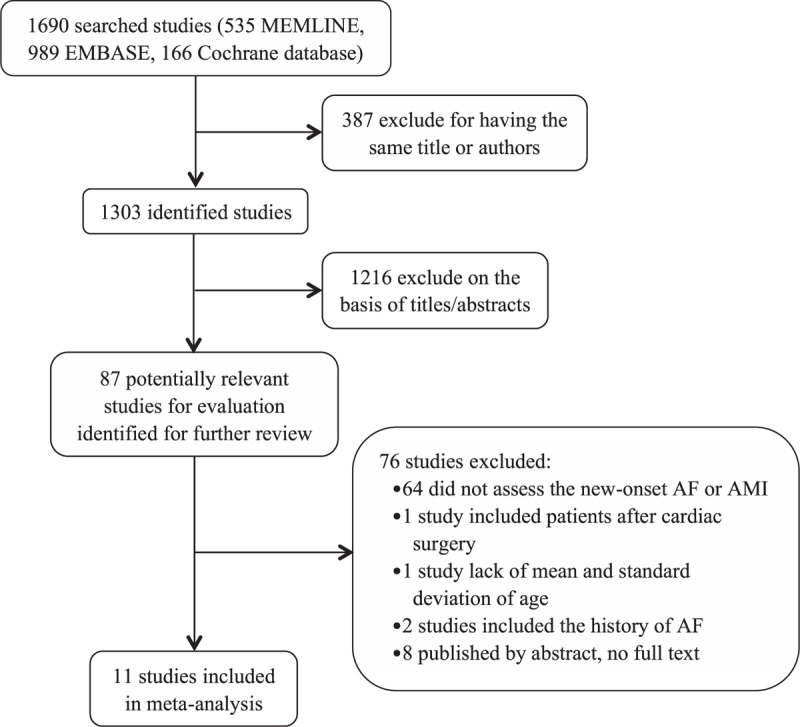
Flow diagram of the trial-selection process. AF = atrial fibrillation, AMI = acute myocardial infarction.

From the initial 1690 studies, 11 were included in the meta-analysis (Fig. 1).^[7,8,10,12–19]^ As a result, 9570 patients were involved in our analysis: 804 patients in AF group and 8766 patients in without AF group. The NOS for assessing the quality of the 11 studies is shown in Table 1 and the scores ranged from 6 to 8. Table 1 presents the characteristics of each study. The mean age of patients in the included studies ranged from 58 to 79 years and the rate of NOAF ranged from 4.8% to 20.7%.

**Table 1:**
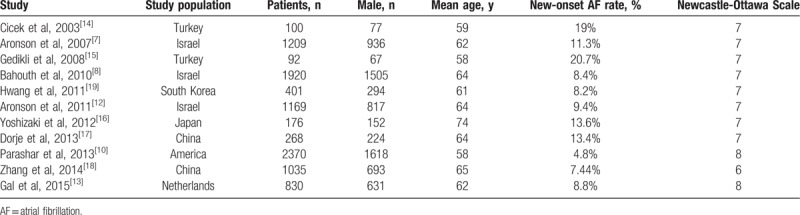
Characteristics of the 11 studies included in the meta-analysis.

### 3.2 Quantitative data synthesis

**Figure 2:**
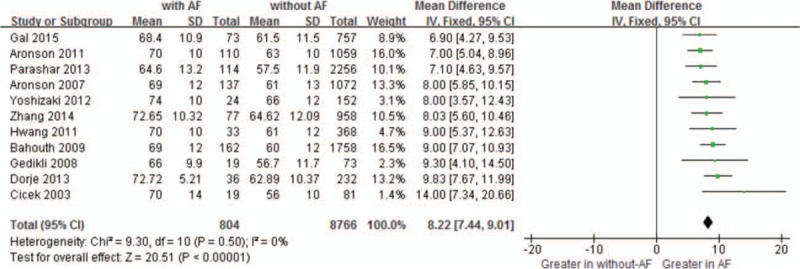
Comparison of age between AF and without AF groups. AF = atrial fibrillation, CI = confidence interval.

**Figure 3:**
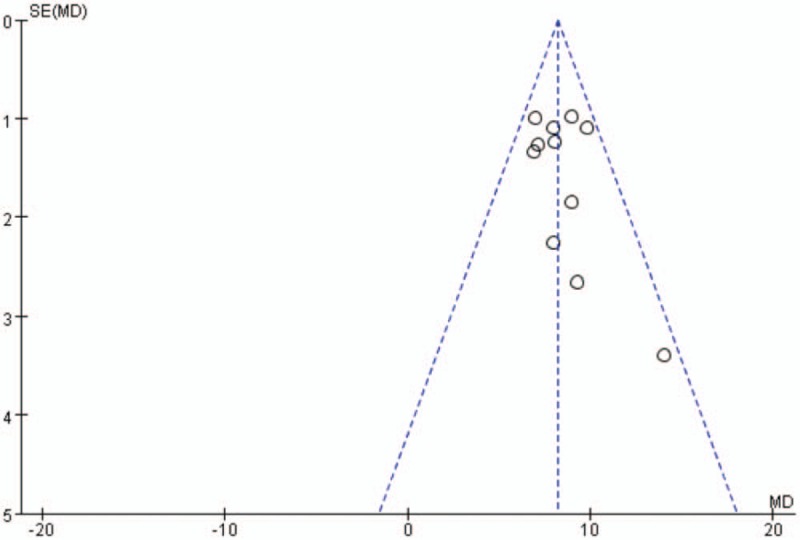
Funnel plot of the 11 included studies. MD = mean difference, SE = standard error.

Overall, there was a significant positive association between age or HR and NOAF in patient with AMI. As shown in Figure 2, the MD in age between the patients with, and those without NOAF was 8.22 units (95% CI: 7.44–9.01), test for overall effect *z* score = 20.51 (*P* < .00001, *I*^2^ = 0%). However, an asymmetric funnel plot shows the possible existence of publication bias (Fig. 3). Because of the small sample size, we cannot explain the exact cause of heterogeneity in our meta-analysis.

**Figure 4:**
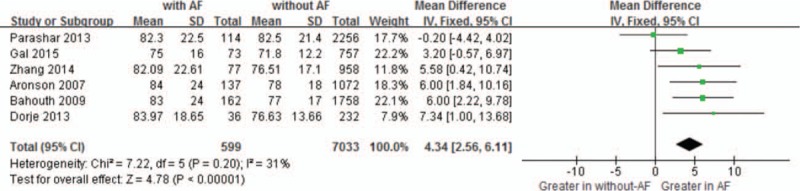
Comparison of HR between AF and without AF groups. AF = atrial fibrillation, CI = confidence interval, HR = heart rate.

**Figure 5:**
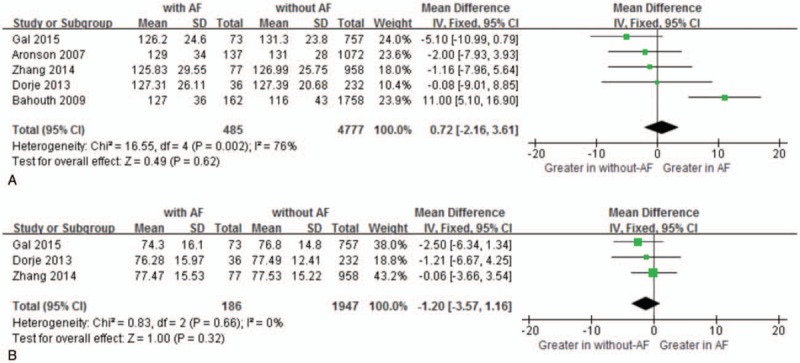
Comparison of SBP (A) and DBP (B) levels between AF and without AF groups. AF = atrial fibrillation, CI = confidence interval, DBP = diastolic blood pressure, SBP = systolic blood pressure.

Moreover, the MD in a subgroup analysis for HR levels between the patients with, and those without NOAF was 4.34 units (95% CI: 2.56–6.11), test for overall effect *z* score = 4.78 (*P* < .00001, *I*^2^ = 31%) (Fig. 4). Besides, the MD in a subgroup analysis for SBP levels between the patients with, and those without NOAF was 0.72 units (95% CI: −2.16 to 3.61), test for overall effect *z* score = 0.49 (*P* = 0.62, *I*^2^ = 76%) (Fig. 5A). The heterogeneity test showed that there were significant differences between individual studies (*P* = 0.002; *I*^2^ = 76%). However, we failed to perform sensitivity analyses to identify the origin of this heterogeneity. The MD in a subgroup analysis for DBP levels between the patients with, and those without NOAF, was −1.20 units (95% CI: −3.57 to 1.16), test for overall effect *z* score = 1.00 (*P* = 0.32, *I*^2^ = 0%) (Fig. 5B).

## 4 Discussion

Present meta-analysis demonstrated that patients who were older were associated with NOAF occurrence after AMI, and also found that increased HR levels on admission were related to greater risk of NOAF following AMI. Furthermore, we also report that there was no relation to blood pressure on admission and NOAF in AMI. Thus, our results may have important clinical implications with adding age and HR into the evaluation tools for risk stratification of NOAF in AMI.

There is no doubt that NOAF gives rise to worse outcomes in AMI patients.^[1–5]^ Hence, it is important to understand the risk stratification of NOAF clearly. However, although plenty of studies have attempted to determine predictors for the occurrence of NOAF in the setting of AMI, the exact mechanisms remain unclear. For previous studies,^[6–13,17,18]^ risk factors for the development of new-onset AF included age, female sex, obesity, Killip class or heart failure, CHADS2 score, creatinine kinase, C-reactive protein, N-terminal pro-brain natriuretic peptide, levels of left ventricular ejection fraction and left atrium diameter. To the best of our knowledge, present meta-analysis is the first study to directly assess the impact of age and HR on NOAF in patients with AMI.

In summary, our meta-analysis demonstrates that older and increased HR levels on admission are related to greater risk of NOAF following AMI. As well known, advanced age is associated with greater prevalence and severity of coronary artery disease and higher risk of ischemic complications and mortality.^[20,21]^ Older patients often carried more co-morbidities, so it was not difficult to understand that old age was a major predisposing factor for the development of AF. HR is an easily and ubiquitously collected vital sign at every clinical patient encounter, and is associated with increased cardiovascular risk in the general population.^[22–29]^ Evidence also showed that admission HR values could independently predict mortality in patients with AMI.^[20,30–32]^ Benjamin et al have demonstrated that increasing HR >65 bpm was associated with worse outcomes, including all-cause and cause-specific mortality, as well as adverse cardiovascular events in patients with AF.^[33]^ HR variability is controlled by a balance between sympathetic and parasympathetic systems, and persistently high resting HRs are seen in stressful situations, chronic illness, and physical inactivity.^[22]^ Several studies indicated that rate control was conducive to reduce cardiovascular morbidity and mortality^[34–36]^; rate control has therefore been adopted as the front-line therapy in many patients with AF.^[20]^ Moreover, beyond our expectation, this meta-analysis found that admission SBP and DBP were not associated with NOAF in AMI. However, because of the small sample size, the result of blood pressure and NOAF in our analysis should be interpreted cautiously.

Several potential limitations of the present meta-analysis should be mentioned. First, although we have collected all the eligible studies, the sample size of the included studies was not large enough. Second, our analysis was based on observational studies, which may result in increasing the potential biases of such studies. Third, present meta-analysis did not include cutoff values about age or HR because the included studies did not have cutoff value data to use. Finally, all included studies were not directly evaluating the relations of age or HR and NOAF, so the potential confounders might have not entirely eliminated.

## 5 Conclusions

In conclusion, our meta-analysis demonstrated that older age and increased HR levels on admission are related to greater risk of NOAF following AMI, and there were no relation of blood pressure and NOAF in AMI.

## Author contributions

**Data curation:** Jing He.

**Methodology:** Xiu-Hong Lu.

**Writing – original draft:** Jing He.

**Writing – review & editing:** Jing He, Yi Yang, Gui Zhang.

## Supplementary Material

Supplemental Digital Content
