# Supplementary material for: Clinical risk factors for new-onset atrial fibrillation in acute myocardial infarction: A systematic review and meta-analysis
Source: Medicine (Baltimore). 2019 Jun 28;98(26):e15960. doi: 10.1097/MD.0000000000015960 (PMC6616519; doi:10.1097/MD.0000000000015960)
Supplement: Supplemental Digital Content [file medi-98-e15960-s001.docx]

**Supplemental Tables 1. Newcastle-Ottawa Quality Assessment Scale–cohort studies**

| Variable | Possible Response |
| --- | --- |
| **Selection** |  |
| 1.Representativeness of the exposed cohort | a)truly representative of the average ________ (describe) in the community*  b)somewhat representative of the average _______ in the community*****  c) selected group of users eg nurses, volunteers  d) no description of the derivation of the cohort |
| 2.Selection of the non-exposed cohort | a) drawn from the same community as the exposed cohort*  b) drawn from a different source  c) no description of the derivation of the non-exposed cohort |
| 3. Ascertainment of exposure | a) secure record (eg surgical records) *****  b) structured interview *****  c) written self report  d) no description |
| 4.Demonstration that outcome of interest was not present at start of study | a) yes*****  b) no |
| **Comparability** |  |
| Comparability of cohorts on the basis of the design or analysis | a) study controls for _____ (select the most important factor) *****  b) study controls for any additional factor (This criteria could be modified to indicate specific control for a second important factor.) ***** |
| **Outcome** |  |
| 1. Assessment of outcome | a) independent blind assessment*****  b) record linkage *****  c) self report  d) no description |
| 2.Was follow-up long enough for outcomes to occur | a) yes (select an adequate follow up period for outcome of interest) *****  b) no |
| 3. Adequacy of follow up of cohorts | a) complete follow up - all subjects accounted for *****  b) subjects lost to follow up unlikely to introduce bias - small number lost->__% (select an adequate%) follow up, or description provided of those lost) *****  c) follow up rate <__% (select an adequate %) and no description of those lost  d) no statement |

**Note:** A study can be awarded a maximum of one star for each numbered item within the Selection and Outcome categories. A maximum of two stars can be given for Comparability

**Supplemental Tables 2. Newcastle-Ottawa Quality Assessment Scale–case control studies**

| Variable | Possible Response |
| --- | --- |
| **Selection** |  |
| 1. Is the case definition adequate? | a) yes, with independent validation*  b) yes, eg record linkage or based on self reports  c) no description |
| 2. Representativeness of the cases | a) consecutive or obviously representative series of cases*  b) potential for selection biases or not stated |
| 3. Selection of Controls | a) community controls *  b) hospital controls  c) no description |
| 4. Definition of Controls | a) no history of disease (endpoint)*  b) no description of source |
| **Comparability** |  |
| Comparability of cohorts on the basis of the design or analysis | a) study controls for _____ (select the most important factor) *****  b) study controls for any additional factor (This criteria could be modified to indicate specific control for a second important factor.) ***** |
| **Exposure** |  |
| 1. Ascertainment of exposure | a) secure record (eg surgical records)*  b) structured interview where blind to case/control status*  c) interview not blinded to case/control status  d) written self report or medical record only  e) no description |
| 2. Same method of ascertainment for cases and controls | a) yes *****  b) no |
| 3. Non-Response rate | a) same rate for both groups*  b) non respondents described  c) rate different and no designation |

**Note:** A study can be awarded a maximum of one star for each numbered item within the Selection and Exposure categories. A maximum of two stars can be given for Comparability.
